# Association of the affordable care act with racial and ethnic disparities in uninsured emergency department utilization

**DOI:** 10.1186/s12913-023-10168-5

**Published:** 2023-11-25

**Authors:** Benjamin Ukert, Theodoros V. Giannouchos

**Affiliations:** 1 Department of Health Policy and Management, Texas A&M University, 212 Adriance Lab Road, 1266 TAMU, College Station, 77843-1266 USA; 2 Department of Health Policy and Organization, The University of Alabama at Birmingham, 1665 University Boulevard, Birmingham, AL 35233 USA

**Keywords:** Racial/ ethnic, Disparities, Emergency departments, Affordable care act, Health care access, Health insurance

## Abstract

**Background:**

Disparities in uninsured emergency department (ED) use are well documented. However, a comprehensive analysis evaluating how the Affordable Care Act (ACA) may have reduced racial and ethnic disparities is lacking. The goal was to assess the association of the ACA with racial and ethnic disparities in uninsured ED use.

**Methods:**

This study used data from the Healthcare Cost and Utilization Project (HCUP) State Emergency Department Databases (SEDD) for Georgia, Florida, Massachusetts, and New York from 2011 to 2017. Participants include non-elderly adults between 18 and 64 years old. Outcomes include uninsured rates of ED visits by racial and ethnic groups and stratified by medical urgency using the New York University ED algorithm. Visits were aggregated to year-quarter ED visits per 100,000 population and stratified for non-Hispanic White, non-Hispanic Black, and Hispanic non-elderly adults. Quasi-experimental difference-in-differences and triple differences regression analyses to identify the effect of the ACA and the separate effect of the Medicaid expansion were used comparing uninsured ED visits by race and ethnicity groups pre-post ACA.

**Results:**

The ACA was associated with a 14% reduction in the rate of uninsured ED visits per 100,000 population (from 10,258 pre-ACA to 8,877 ED visits per 100,000 population post-ACA) overall. The non-Hispanic Black compared to non-Hispanic White disparity decreased by 12.4% (-275.1 ED visits per 100,000) post-ACA. About 60% of the decline in the Black-White disparity was attributed to disproportionate declines in ED visit rates for conditions classified as not-emergent (-93.2 ED visits per 100,000), and primary care treatable/preventable (-64.1 ED visits per 100,000), while the disparity in ED visit rates for injuries and not preventable conditions also declined (-106.57 ED visits per 100,000). All reductions in disparities were driven by the Medicaid expansion. No significant decrease in Hispanic-White disparity was observed.

**Conclusions:**

The ACA was associated with fewer uninsured ED visits and reduced the Black-White ED disparity, driven mostly by a reduction in less emergent ED visits after the ACA in Medicaid expansion states. Disparities between Hispanic and non-Hispanic White adults did not decline after the ACA. Despite the positive momentum of declining disparities in uninsured ED visits, disparities, especially among Black people, remain.

**Supplementary Information:**

The online version contains supplementary material available at 10.1186/s12913-023-10168-5.

## Introduction

The aims of the Patient Protection and Affordable Care Act (ACA) were to expand health insurance coverage and to improve access to healthcare services. The primary components of the ACA, the subsidized private health insurance marketplace and the expansion of Medicaid eligibility, increased health insurance coverage and improved access to outpatient care, including preventive, diagnostic, and surgical services [1, 2]. The ACA also reduced cost-barriers to care, and improved care continuity, health outcomes and quality-of-care markers [1, 3–6].

Prevailing racial and ethnic disparities in health insurance coverage have long characterized the US healthcare system [7]. Historically, these disparities contributed to poorer access to care and worse health outcomes for minorities [8]. The ACA was associated with narrowing the gap in health disparities among racial and ethnic minorities, such as having a personal doctor and foregoing care due to cost [8–18]. Whether these improvements translate to the use of emergency departments (EDs) are currently not well understood. The existing evidence on the association of the ACA and ED visits is mixed and suggests that ED visits may not have changed much after the enactment of the ACA, although recent findings suggest that ED visits for less emergent conditions decreased in some states that expanded Medicaid compared to states that did not [19–23].

Opportunities to reduce ED care dependence for minorities remain a viable policy path. In 2010, minorities accounted for 40% of ED visits, even though they only represented 28% of the population [24, 25]. Higher rates of uninsurance, social risks and needs, and legal mandates on EDs are drivers of disparities in ED care [26–29]. This suggests a huge potential to redirect individuals to more cost-effective outpatient settings [30, 23, 31–34]. A recent study documented that the Medicaid expansions were associated with decreased disparities in preventable hospitalizations and ED visits between non-Hispanic Black and White nonelderly adults [35]. However, to our knowledge, no study has explored the association of the ACA on racial and ethnic disparities in uninsured ED visits and particularly by medical urgency.

In this study we estimate the association of the ACA with changes in racial and ethnic health disparities in uninsured ED visit rates and stratified by medical urgency. Building on previous work that found decreases in the shares of ED visits for the previously uninsured and decreases in ED visits for less medically urgent conditions, our study extends the current literature by providing evidence on the differential impact of the ACA on racial and ethnic disparities in uninsured ED use. We hypothesized that the disproportionately larger gains in health insurance coverage after the ACA for minorities might have led to reductions in ED visits by the uninsured and thus narrowed racial and ethnic disparities. We further expected that any closing in disparity would be attributed mostly to less medically urgent conditions which could be substituted with outpatient care [5, 31, 23]. Our findings are important to evaluate policies that enhance access to care, and to offer insight on how potentially substitutable or preventable ED visits were affected after the ACA for racial and ethnic groups.

## Methods

### Data and measures

We conducted a retrospective data analysis using the 2011 to 2017 Healthcare Cost and Utilization Project (HCUP) State Emergency Department Databases (SEDD) for four states (Georgia, Florida, Massachusetts, and New York) which account for about one-fifth of the US population [36]. The data cover three years before the ACA’s insurance expansions (2011–2013) and four years after (2014–2017) and further allowed us to stratify states by Medicaid expansion status (Massachusetts and New York expanded; Georgia and Florida did not expand). The HCUP SEDD are longitudinal, administrative, secondary databases which include sociodemographic, clinical, and reimbursement information for ED visits. Our main sample included only ED visits that did not result in hospital admissions across all general and acute state hospitals statewide. We excluded hospitalizations, which make up only about 7% of all ED visits, to study how ambulatory care sensitive ED visits, which are more likely to be treatable in other healthcare settings may be affected by the insurance expansions. We also restricted our analysis to non-elderly adults ages 18 to 64, because these individuals were targeted by the main health insurance coverage provisions of the ACA that went into effect in 2014 [5, 11]. Similar to previous work, we included ED visits for non-Hispanic White (henceforth White), non-Hispanic Black (henceforth Black), and Hispanic adults, since these three groups account for 92% of the US population, and because only few visits occurred for other race and ethnicity groups [12].

The outcomes of interest were the overall rates of uninsured ED visits per 100,000 state population 18–64 years of age during the study period by race and ethnicity (Hispanic, Black, and White). Population numbers by race and ethnicity were obtained from publicly available data [37, 38]. Insurance status for each ED visit was identified using the primary payer source on each ED visit similar to previous work, and then aggregated to the year-quarter level for each state, resulting in racial and ethnic specific uninsurance rates in each year-quarter period for each state [34]. Our secondary outcomes included rates of uninsured ED visits classified by medical urgency and race and ethnicity. We used the updated version of the New York University (NYU) ED algorithm to assign probabilities to each ED visit by medical urgency [39]. The algorithm assigns probabilities between 0 and 100% based on the primary diagnosis across four categories—Emergent-not preventable/avoidable, Emergent but preventable, Emergent but primary care treatable, and Not Emergent. The algorithm also separately identifies diagnoses related to injury, mental health, alcohol and drug use. To reflect medical urgency, we created four probabilistic adjusted categories for each ED visit, namely 1) Not preventable/Injuries, 2) Preventable/Primary Care Treatable, 3) Not Emergent, 4) Substance use/Mental health. Some diagnoses remained unclassified, but represented only about 12% of all ED visits. We used primary diagnostic codes based on the International Statistical Classification of Diseases and Related Health Problems, 9^th^ and 10^th^ Revisions codes.

We obtained information on individuals’ sociodemographic and clinical characteristics. Sociodemographic variables included sex, age group, income quartile at the area of residence, based on the zip code, and rurality. Individual level information was aggregated to the race-state-year-quarter level and we calculated the share of being female, age-group shares (percentages 18 to 34, 35 to 44, and 55 to 64), income share (percentage in lowest quartile), and shares of those living in urban–rural areas (large and small metropolitan areas) based on the individuals seen in the ED. All diagnostic codes available at each ED visit were used to construct these clinical measures. Additional time-varying variables at the state and race and ethnicity levels included publicly available poverty and unemployment rates [40, 41].

### Study design

We used a quasi-experimental regression design that includes difference-in-differences and difference-in-differences-in-differences interactions to evaluate disparities in uninsured ED visit rates for Hispanic and Black adults relative to White adults after the ACA. We compared disparities between the pre-ACA implementation period (2011–2013) and the post-ACA implementation period (2014–2017) by Medicaid expansion status. The difference-in-differences coefficient identifies the national component of the ACA which were experienced by all states, while the triple differences effect measures the separate impact of the Medicaid expansion on uninsured ED visit rates. The unit of analysis was the rate of uninsured ED visits per 100,000 for each state at the year-quarter level by race and ethnic groups.

### Statistical analysis

We first conducted a descriptive analysis to characterize ED visits by the uninsured population in the pre-ACA and post-ACA periods. We then classified our sample into three groups (Hispanic, Black, and White) and compared trends in uninsured ED visit rates for Black and Hispanic adults relative to White adults. Formally, we estimated the association of the ACA and the Medicaid expansion implementations with changes in racial and ethnic disparities using linear regression models, controlling for sociodemographic and state-level covariates mentioned above and state and year-fixed effects, as described above. This approach enabled us to compare how racial and ethnic disparities changed after 2014 due to the national component of the ACA and the changes attributed to the Medicaid expansion. We also identified the total effects of the ACA (ACA and Medicaid expansion) on racial and ethnic disparities using linear combinations of the two separate estimates (see Additional file 1 for detail provided online). Similar approaches to identify the two major components of the ACA have been used before [11].

The parallel trends assumption requires common trends in outcomes for the treatment groups (Black and Hispanic) compared to the control group (White) in the pre-policy period [42]. We indirectly tested the parallel trends assumption by examining uninsured ED visit rates in the pre-ACA period (2011–2013). Specifically, we limited the sample to 2011–2013 and estimated coefficients for each quarter from 2011 to 2013 (with the first quarter of 2011 as the reference group) for the treatment groups relative to the comparison group. To do so, we interacted the difference-in-difference Hispanic or Black indicator variable with each quarter-year indicator between 2011 and 2013, keeping all other control variables as described above. We also interacted the Medicaid expansion variable with the Hispanic or Black indicator variable with each quarter-year between 2011 and 2013. In this regression, one would expect to find similar trends in ED visits for Hispanic and Black adults relative to White adults through statistically insignificant coefficients on all interaction terms. Across all models, robust standard errors were used.

Data management was conducted using SAS version 9.4 (SAS Institute) and all analyses were conducted using Stata version 17.0 (StataCorp). The study was determined to be non-human subjects research and was approved by the [blinded for review] University Institutional Review Board.

## Results

Table 1 provides an overview of ED visits and the trends in ED visits before and after the ACA overall and by the uninsured. The number of total ED visits increased from the pre- to the post-ACA period from 10.4 million to 10.8 million per year, on average, while the number of uninsured ED visits decreased from 2.9 million per year in the pre-ACA period to 2.5 million in the post-ACA period, on average. Women, individuals ages 18 to 34, residents of large metropolitan areas, and those residing at locations with the lowest income accounted for a larger share of uninsured ED visits in the pre- and post-ACA period. In terms of rates, the overall uninsured ED visit rate declined by 14% (from 10,258 to 8,877 ED visits per 100,000 population) from the pre to the post-ACA period (Table 1). Additional file 1: Figure S1 displays the declines by ED visit type. At the same time, the share of Medicaid insured visits increased from 37.5% in 2011–2013 to 39.6% in 2014–2017 (see Additional file 1: Table S1 for additional detail provided online).

**Table 1:** Descriptive Statistics for all ED Visits by Uninsured non-elderly Adults from 2011 to 2017

	**Overall**	**Pre-ACA**	**Post-ACA**
	**(2011–2017)**	**(2011–2013)**	**(2014–2017)**
**Number of ED visits (million)**			
Total (in million)	74.6	31.2	43.4
Total annual (average in million)	10.7	10.4	10.8
Total quarterly rates (average per 100,000 population)	34,124.2	33,374.0	34,683.9
**Number of uninsured ED visits**			
Total (in million)	18.7	8.7	10.0
Total annual (average in million)	2.7	2.9	2.5
Total quarterly rates (average per 100,000 population)	9469.3	10,257.6	8876.6
**Characteristics of uninsured ED visits**			
**Race and ethnicity** (%)			
Non-Hispanic White	46.8	48.3	46.0
Non-Hispanic Black	36.7	35.6	38.0
Hispanic	16.5	16.1	17.0
**Gender** (%)			
Female	59.2	59.2	59.2
Male	40.8	40.8	40.8
**Age groups** (%)			
18 to 34	49.0	50.0	48.2
35 to 44	20.9	20.9	20.9
45 to 54	18.5	18.4	18.5
55 to 64	11.7	10.7	12.4
**Income quartiles (median of patients’ ZIP code)** (%)			
1^st^ (lowest)	41.5	41.5	41.5
2^nd^	26.6	26.2	26.9
3^rd^	19.2	19.4	19.1
4^th^ (highest)	12.7	12.9	12.5
**Area of residence** (%)			
Large metropolitan	65.8	65.1	66.3
Small metropolitan	25.1	25.5	24.8
Non-metropolitan	9.1	9.4	8.9
**Classification by medical urgency** (%)			
Not Preventable/Injuries	30.1	30.8	29.5
PCT/Preventable	27.7	28.1	27.4
Not Emergent	23.8	24.7	23.1
Mental Health/Substance Use	5.5	5.1	5.7
**Elixhauser comorbidities**			
Average (standard deviation)	0.5 (0.08)	0.4 (0.07)	0.5 (0.07)
Most prevalent (%)			
Hypertension	12.7	11.3	13.8
Chronic obstructive pulmonary	7.1	6.4	7.7
Disease			
Diabetes	5.9	5.2	6.4
Depression	2.9	2.7	3.0
Fluid and electrolyte disorders	1.9	1.8	1.9
Cardiac arrythmia	1.5	1.4	1.7

Table displays selected ED and socioeconomic averages for the full sample period (2011–2017), and stratified by pre-and post-ACA period

**Fig. 1:**
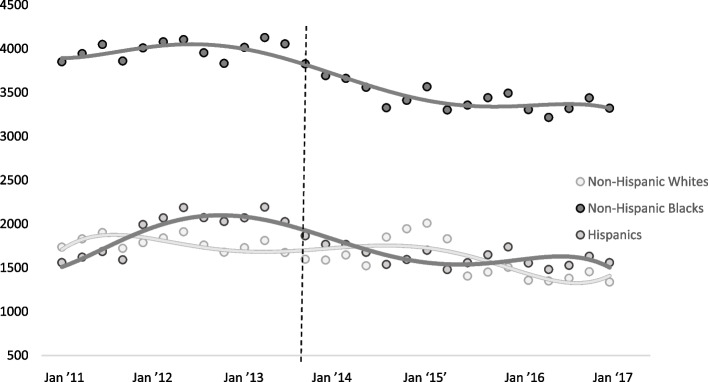
Trend in Quarterly Uninsured ED Visit Rates Before and After the ACA by Race and Ethnicity. *Notes*: HCUP data from 2011–2017 displaying the share of nonelderly uninsured ED visit rates per 18–64 year of age 100,000 population by racial and ethnic groups before and after the implementation of the insurance expansion in January of 2014

Stratifying uninsured ED visits across racial and ethnic groups provided evidence of differences in ED use (see Additional file 1: Table S2 for additional detail provided online). Compared to White adults, Black and Hispanic adults had disproportionately higher rates of uninsured ED visits, larger shares of uninsured visits among 18 to 34 year-olds, residents in large metropolitan areas, and those in areas with the lowest incomes. However, all racial and ethnic groups experienced a declining trend in uninsured ED visit rates after the ACA implementation (Fig. 1). The average quarterly decline in the uninsured ED visit rate from the pre- to the post-ACA period was 11.5% among White adults (-819 ED visits per 100,000), 15.3% for Hispanic adults (-1,178 ED visits per 100,000), and 13.5% for Black adults (-2,152 ED visits per 100,000).

Table 2 presents the unadjusted and adjusted regression results describing the change in disparities in uninsured ED visit outcomes by racial and ethnic groups relative to White adults from the pre-ACA period to the post-ACA period. For most outcomes, we observed large disparities in uninsured ED visit rates in the pre-ACA period. The ACA was significantly associated with a reduction in disparities in the uninsured ED visit rate for Black adults compared to White adults by 12.4% (-275.1 ED visits per 100,000; 95% CI, -482.3 to -67.9). About half of this decrease in the Black-White gap was attributable to statistically significant declines in uninsured ED visit rates classified as primary care treatable or preventable (-64.1 ED visits per 100,000; 95% CI, -122.7 to -5.5) and as not emergent (-93.2 ED visits per 100,000; 95% CI, -149.5 to -36.9). The remaining reduction emerged from not preventable or injury-related ED visit rates (-106.5 ED visits per 100,000; 95% CI, -175.9 to -37.1). No significant decrease in Hispanic-White disparity was observed. Additional file 1: Table S3 displays the same regression results excluding Massachusetts from the treatment group and provides qualitatively similar conclusions (see Additional file 1: Table S3 for additional detail provided online).

**Table 2:** Difference-in-Differences and Difference-in-Differences-in-Differences Estimates of the Association of the ACA With the Uninsured ED Visit Rates by Race and Ethnicity

	**Pre-ACA**	**Post-ACA**	**Unadjusted**	**Adjusted**	**Adjusted** **ACA Only**	**Adjusted Medicaid Expansion Only**
**Overall uninsured ED visits**						
White	1781.1	1576.3				
Black	3989.5	3451.5	-333.2	-275.1^a^	135.2	-410.3^a^
Hispanic	1924.2	1629.7	-89.7	-94.2	183.5	-277.7
**Not Preventable/Injuries**						
White	583.4	484.0				
Black	1096.8	912.5	-84.9	-106.5^a^	31.3	-137.8^a^
Hispanic	596.0	481.1	-15.5	-32.2	50.0	-82.2
**PCT/Preventable**						
White	503.9	449.2				
Black	1182.4	1001.3	-126.4	-64.1^b^	30.3	-94.4^b^
Hispanic	553.8	463.6	-35.5	-32.2	64.2^b^	-96.4^b^
**Not Emergent**						
White	396.3	327.7				
Black	1099.0	912.1	-118.3	-93.1^a^	44.9	-138.1^a^
Hispanic	480.5	383.0	-28.9	-39.1	43.3	-82.4
**Mental Health/Substance Use**						
White	106.3	102.7				
Black	143.4	139.9	0.1	4.4	19.0^a^	-14.6^b^
Hispanic	83.9	74.1	-6.2	5.6	3.8	1.8

Adjusted estimates were obtained from DD and triple differences coefficients using multivariable ordinary least squares regressions adjusted for sociodemographic and state-level covariates. The combined effect (column 4) displays the impact of the national component of the ACA (column 5) and the Medicaid expansion (column 6). Each row represents estimates from a separate regression model in columns 4–6. Pre-ACA refers to the 2011 to 2013 years and post-ACA to the 2014 to 2017 years. DD: Difference-in-Differences, PCT: Primary Care Treatable

^a^indicates statistically significant at the 1% level

^b^indicates statistically significant at the 5% level

Separating the ACA effect due to the nationwide policies and the Medicaid expansion displayed that reductions in uninsured ED visits were driven by the Medicaid expansion. For all outcomes, the reduction in disparities stemming from the Medicaid expansion was statistically significant and larger in absolute terms than the national ACA component effect. For example, the Medicaid expansion reduced the uninsured ED visit disparities by 410.3 visits per 100,000 (95% CI, -657.9 to -162.7) for Black adults compared to White adults, while the ACA effect independent of the Medicaid expansion was insignificant (135.2, 95% CI, -39.6 to 309.9).

### Event study test

We found strong evidence that supported our identification strategy. Overall, we had 11 pre-ACA year-quarter periods to evaluate trends in visit rates in each of the outcomes. In our event studies results for the total uninsured ED rates, we found no statistically significant coefficients in the pre-policy period for the Hispanic-White and Black-White comparisons (See Additional file 1: Table S4 for additional detail provided online) for the national ACA effect, suggesting similar trends for Hispanic and Black adults compared to White adults. Across all regressions stratifying ED visits by the NYU ED algorithm categories, we observed a low rejection rate of the null hypothesis; 4 rejections in a total of 88 coefficients for the Hispanic and Black disparity regressions (see Additional file 1: Table S5 for additional detail provided online). Analysis for the triple difference Medicaid expansion coefficients displayed similarly low rejection rate for all 5 outcomes (9 rejections out of 110 coefficients; 8.2%).

## Discussion

The implementation of the ACA was associated with reductions in uninsured ED visits rates for all racial and ethnic groups and decreased disparities in uninsured ED visit rates for Black adults relative to White adults. The decrease in disparities was concentrated across three types of ED visits; not preventable and injury-related visits, primary care treatable and preventable visits, and non-emergent visits, and was driven by Medicaid expansion states relative to non-Medicaid expansion states. At the same time, we did not observe a decline in disparities in Hispanic compared to White uninsured ED visit rates even in Medicaid expansion states.

Our findings are in-line with recent work documenting decreases in disparities for non-Hispanic Black and White adults for preventable hospitalizations and ED visits but not between Hispanic and non-Hispanic White adults [35]. The closing disparities gap for non-emergent, primary care treatable and preventable conditions, documented in our and a previous study, suggests better access to outpatient care once many of the previously uninsured obtained health insurance coverage through the provisions of the ACA [35]. This notion is supported by the fact that the ACA reduced racial and ethnic disparities in health insurance coverage, and increased access to primary care for low income individuals and minorities [9–13, 43, 44]. Outpatient interactions enable continued disease management, thus averting adverse outcomes which can result in acute and after hour ED visits [1, 2]. Of note is that the gap decreased due to the Medicaid expansion, which can be expected given that the ACA Medicaid expansions have been associated with increased access to care particularly for minorities [4, 12]. Despite this progress, ED care remains a regular access point for the remaining uninsured [45, 46]. Further, Black and Hispanic adults remain more likely to be uninsured than their White counterparts and, as our study also indicates, exhibit the highest rates of ED use [9].

Our findings further indicated that a large share of ED visits continues to be preventable and treatable with timely outpatient care, displaying untapped opportunities to reduce ED visits, especially among minorities. Existing interventions to mitigate adverse effects of social determinants of health on ED patients that were implemented in the United States did not focus on the intersectionality between race and ethnicity and insurance status to reduce disparities for minorities and underrepresented groups [47]. As our findings suggest, future work should focus on directly addressing this overlap to rectify disparities in access to regular care and curtail non-emergent ED visits by the uninsured. Reduced uninsured ED visits have also positive implications for hospital revenue and profit, while also potentially improving state budget flexibility with lower levels of uncompensated care funds required to be transferred to hospitals [48].

This work is not without limitations. The HCUP data uses hospital reported race and ethnicity data, and this measure may suffer from mismeasurement. However, evidence suggests that race and ethnicity information in discharge data is generally valid for major racial and ethnic groups analyzed in this study [49]. We also did not include other minorities with relatively little ED use in our study, such as Asian Americans and Native Americans, which may limit the generalizability of our findings. We further note that a share of the reductions in ED visits might be related to adverse selection, with those who remained uninsured being healthier in the post-ACA period and thus less likely to seek ED care anyway. However, the analytical approach used in this study, which explores differences in disparities between racial and ethnic groups in the pre-and post-ACA period, should not be biased for this reason given the overall similarity of comorbidities across racial and ethnic groups in the post-ACA period. We also note the NYU ED algorithm classified ED visits using discharge diagnosis, rather than presenting complaints, which might underestimate patient- and provider-perceived urgency at the time of the arrival to the ED. Moreover, the NYU algorithm did not enable us to classify a remaining 12% of ED visits. Our study may also suffer from confounders that differentially affect race and ethnicity after the ACA, such as changes in hospital care delivery systems, though these impacts should be minimized given that our event study did not display differential pre-trends. Finally, our analysis is limited to four large states and may not be generalizable to all 50 states. Nevertheless, our results are broadly consistent with the literature that has shown limited changes in total ED visits and reductions in uninsured ED visits after the ACA across the US [3, 29, 30].

## Conclusion

In this study, the ACA was associated with a narrowing disparity in the Black-White uninsured ED visit rate, and the decrease was driven by Medicaid expansion states compared to non-expansion states. However, disparities remain, particularly in states that have not expanded Medicaid, and policy is needed to address and close remaining gaps for minority groups.

### Supplementary Information

**Additional file 1: Figure S1.** Trend in the Quarterly Uninsured ED Visit Rates Before and After the ACA by Medical Urgency using the NYU ED algorithm. **Table S1.** Descriptive Statistics for All Insured non-elderly ED Visits from 2011 to 2017. **Table S2.** Descriptive Statistics for all ED Visits by Uninsured non-elderly Adults from 2011 to 2017 Stratified by Race and Ethnicity. **Table S3.** Difference-in-Differences and Difference-in-Differences-in-Differences Estimates of the Association of the ACA With the Uninsured ED Visit Rates by Race and Ethnicity (Excluding Massachusetts from the Treatment Group). **Table S4.** Indirect Test of the Parallel Trends Assumption in the pre-ACA Implementation Period for all uninsured ED Visits Rates by Racial and Ethnic Groups. **Table S5.** Indirect Test of the Parallel Trends Assumption in the pre-ACA Implementation Period for Uninsured ED Visits Rates by Racial and Ethnic Groups Classifying Visits using NYU Algorithm. 

## Data Availability

The datasets used and analyzed in the study are available from AHRQ [https://www.hcup-us.ahrq.gov/]. Data cannot be shared by the authors because dataset can only be obtained after the submission of a study proposal and payment.

## Abbreviations

ACA: Affordable Care Act
ED: Emergency Department
HCUP: Healthcare Cost and Utilization Project
SEDD: State Emergency Department Databases
NYU: New York University
