# Supplementary material for: Association of the affordable care act with racial and ethnic disparities in uninsured emergency department utilization
Source: BMC Health Serv Res. 2023 Nov 25;23:1302. doi: 10.1186/s12913-023-10168-5 (PMC10676572; doi:10.1186/s12913-023-10168-5)
Supplement: Supplementary file 1 — Additional file 1: Figure S1. Trend in the Quarterly Uninsured ED Visit Rates Before and After the ACA by Medical Urgency using the NYU ED algorithm. Table S1. Descriptive Statistics for All Insured non-elderly ED Visits from 2011 to 2017. Table S2. Descriptive Statistics for all ED Visits by Uninsured non-elderly Adults from 2011 to 2017 Stratified by Race and Ethnicity. Table S3. Difference-in-Differences and Difference-in-Differences-in-Differences Estimates of the Association of the ACA With the Uninsured ED Visit Rates by Race and Ethnicity (Excluding Massachusetts from the Treatment Group). Table S4. Indirect Test of the Parallel Trends Assumption in the pre-ACA Implementation Period for all uninsured ED Visits Rates by Racial and Ethnic Groups. Table S5. Indirect Test of the Parallel Trends Assumption in the pre-ACA Implementation Period for Uninsured ED Visits Rates by Racial and Ethnic Groups Classifying Visits using NYU Algorithm. [file 12913_2023_10168_MOESM1_ESM.docx]

**Additional file 1**

Our regression model includes difference in difference (DD) and difference-in-differences-in-differences (DDD) components and specified as:

$$y_{ist}= a_{1}+ \beta_{1}{Race}_{i}+ \beta_{2}Post ACA_{t}+ \beta_{3}{Race}_{i}*Post ACA_{t} +\beta_{4}{Medicaid Expansion}_{s}+\beta_{5}{Race}_{i}*{Medicaid Expansion}_{s} + \beta_{6}Post ACA_{t} *{Medicaid Expansion}_{s}+ \beta_{7}{Race}_{i}*Post ACA_{t}*{Medicaid Expansion}_{s}+\gamma X_{ist}+{\zeta State}_{s}+ {\tau Year}_{t}+\varepsilon_{ist}$$

where $y_{ist}$ is one of the uninsured ED outcome variables per 100,000 population for race and ethnic group *i* in state *s* and quarter-year *t*. Each regression compares ED outcomes for either Hispanics versus whites or blacks versus whites, with white being the reference group. Thus, $Race$ is a binary indicator for the race group (Hispanic or black) and $Post ACA$is an indicator variable equal to one beginning in 2014 and zero otherwise; $\beta_{3}$ indicates the effect of ACA implementation on disparities among Hispanics or blacks after 2014 relative to whites. $\beta_{7}$ provides the separate effect of the Medicaid expansion component on disparities among Hispanics or blacks after 2014 relative to whites. *X* is a vector that includes visit-level characteristics, namely gender (percentage female), age-groups (percentages 18 to 34, 35 to 44, and 55 to 64), income (percentages in the lowest quartile), rurality of patients’ residence (percentages of residents in large or small metropolitan areas), and the average Elixhauser comorbidity index, and state-level poverty and unemployment rates for each race and ethnic group in each state quarter-year. We further included state and year fixed effects to control for unobserved state and time specific.

Coefficients from $\beta_{3} and \beta_{7}$ provide estimates on the impact of the change in disparities due to the ACA portion that affects all states (e.g., marketplace etc.) and the Medicaid expansion. We use the combination of the two coefficients to arrive at the “adjusted DD” result for the overall ACA effect displayed in Table 2.

Figure S1: Trend in the Quarterly Uninsured ED Visit Rates Before and After the ACA by Medical Urgency using the NYU ED algorithm


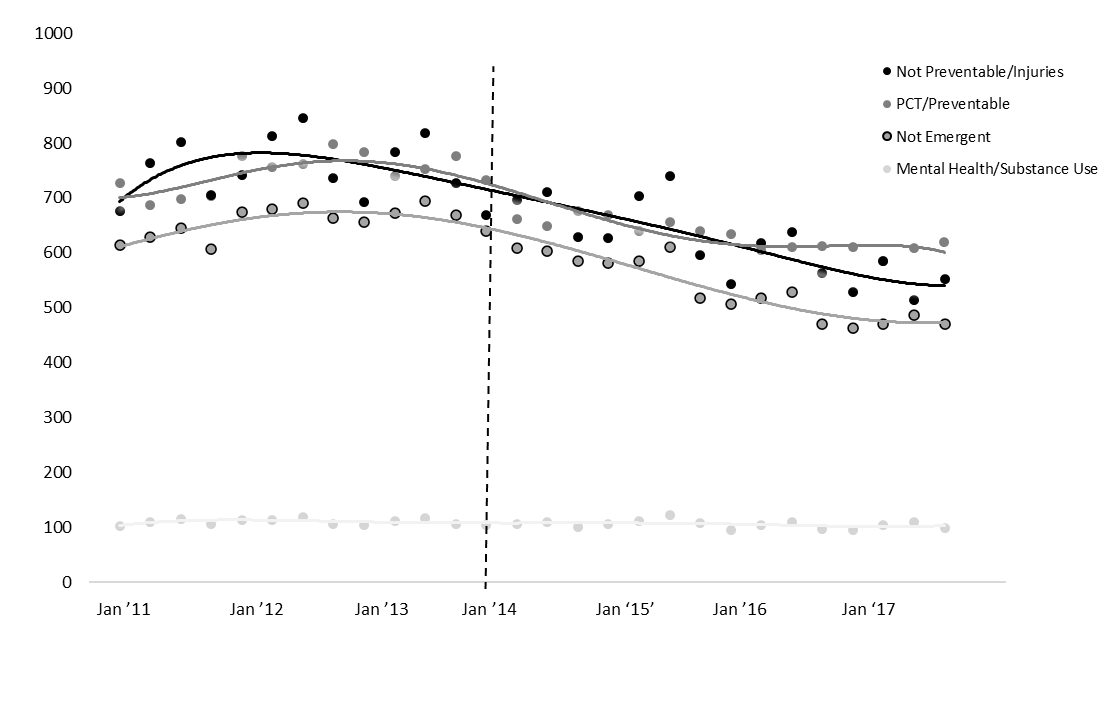


Notes: HCUP data from 2011-2017 displaying uninsured ED visit rates per 100,000 population by NYU ED classification before and after the implementation of the insurance expansion in January of 2014 among non-elderly adults.

Table S1: Descriptive Statistics for All Insured non-elderly ED Visits from 2011 to 2017

Notes: Table displays socioeconomic averages for insured ED visits for the full sample period (2011-2017) and stratified by pre-and post-ACA period.

Table S2: Descriptive Statistics for all ED Visits by Uninsured non-elderly Adults from 2011 to 2017 Stratified by Race and Ethnicity

|  | **Non-Hispanic Whites** | | |  | **Non-Hispanic Blacks** | | |  |  | **Hispanics** |  |
| --- | --- | --- | --- | --- | --- | --- | --- | --- | --- | --- | --- |
|  | **Overall** | **Pre-ACA** | **Post-ACA** | | **Overall** | **Pre-ACA** | **Post-ACA** | | **Overall** | **Pre-ACA** | **Post-ACA** |
| **Number of ED visits (million)** |  |  |  |  |  |  |  |  |  |  |  |
| Total (in million) | 39.0 | 16.7 | 22.3 |  | 23.3 | 9.4 | 13.9 |  | 12.3 | 4.5 | 7.8 |
| Total annual (average in million) | 5.6 | 5.6 | 5.6 |  | 3.3 | 3.1 | 3.5 |  | 1.8 | 1.5 | 2.0 |
| Total quarterly per 100,000 population  (average) | 29369.8 | 28941.7 | 29690.9 |  | 59566.1 | 58169.6 | 60613.6 |  | 31760.7 | 31561.4 | 31910.2 |
| **Number of uninsured ED visits** |  |  |  |  |  |  |  |  |  |  |  |
| Total (in million) | 8.8 | 4.2 | 4.6 |  | 6.9 | 3.1 | 3.8 |  | 3.1 | 1.4 | 1.7 |
| Total annual (average in million) | 1.2 | 1.4 | 1.1 |  | 1.0 | 1.0 | 0.9 |  | 0.4 | 0.5 | 0.4 |
| Share (average %) | 21.0 | 23.5 | 19.1 |  | 24.8 | 27.8 | 22.5 |  | 27.0 | 30.4 | 24.5 |
| Total quarterly per 100,000 population  (average) | 6656.4 | 7124.4 | 6305.2 |  | 14728.4 | 15958.0 | 13806.0 |  | 7023.6 | 7696.8 | 6518.8 |
| **Sociodemographic characteristics of uninsured ED visits** | |  |  |  |  |  |  |  |  |  |  |
| **Gender** (%) |  |  |  |  |  |  |  |  |  |  |  |
| Female | 56.9 | 56.8 | 57.0 |  | 61.0 | 61.3 | 60.8 |  | 59.8 | 59.6 | 59.9 |
| Male | 43.1 | 43.2 | 43.0 |  | 39.0 | 38.7 | 39.2 |  | 40.2 | 40.4 | 40.1 |
| **Age groups** (%) |  |  |  |  |  |  |  |  |  |  |  |
| 18 to 34 | 44.9 | 46.3 | 43.9 |  | 51.2 | 51.8 | 50.8 |  | 50.8 | 51.9 | 49.9 |
| 35 to 44 | 20.4 | 20.5 | 20.3 |  | 20.0 | 19.9 | 20.1 |  | 22.3 | 22.2 | 22.3 |
| 45 to 54 | 20.1 | 20.0 | 20.1 |  | 18.1 | 18.4 | 17.8 |  | 17.2 | 16.8 | 17.6 |
| 55 to 64 | 14.6 | 13.3 | 15.6 |  | 10.7 | 9.8 | 11.4 |  | 9.7 | 9.0 | 10.2 |
| **Income quartiles (median of patients' ZIP code)** (%) | |  |  |  |  |  |  |  |  |  |  |
| 1st (lowest) | 26.4 | 26.7 | 26.3 |  | 52.7 | 52.8 | 52.6 |  | 45.3 | 45.1 | 45.5 |
| 2nd | 29.0 | 28.9 | 29.1 |  | 24.2 | 23.7 | 24.6 |  | 26.6 | 26.0 | 27.0 |
| 3rd | 25.0 | 24.8 | 25.1 |  | 15.7 | 16.3 | 15.3 |  | 17.0 | 17.1 | 16.8 |
| 4th (highest) | 19.5 | 19.6 | 19.5 |  | 7.4 | 7.3 | 7.4 |  | 11.1 | 11.7 | 10.7 |
| **Area of residence** (%) |  |  |  |  |  |  |  |  |  |  |  |
| Large metropolitan | 54.3 | 53.2 | 55.1 |  | 72.9 | 72.1 | 73.4 |  | 70.3 | 69.9 | 70.5 |
| Small metropolitan | 31.7 | 32.2 | 31.3 |  | 21.2 | 21.6 | 21.0 |  | 22.5 | 22.7 | 22.3 |
| Non-metropolitan | 14.0 | 14.6 | 13.6 |  | 5.9 | 6.3 | 5.6 |  | 7.2 | 7.4 | 7.2 |
| **Clinical characteristics of uninsured ED visits** |  |  |  |  |  |  |  |  |  |  |  |
| **Classification by medical urgency** (%) |  |  |  |  |  |  |  |  |  |  |  |
| Not Preventable/Injuries | 32.5 | 33.4 | 31.8 |  | 27.8 | 28.2 | 27.5 |  | 29.9 | 31.0 | 29.1 |
| PCT/Preventable | 26.9 | 27.1 | 26.7 |  | 28.1 | 28.8 | 26.6 |  | 28.2 | 28.6 | 27.9 |
| Not Emergent | 20.8 | 21.9 | 19.9 |  | 26.5 | 27.2 | 25.8 |  | 24.1 | 24.9 | 23.4 |
| Mental Health/Substance Use | 7.4 | 6.9 | 7.7 |  | 4.4 | 4.0 | 4.6 |  | 4.7 | 4.5 | 4.9 |
| **Elixhauser comorbidities** |  |  |  |  |  |  |  |  |  |  |  |
| Average (standard deviation) | 0.5 (0.07) | 0.4 (0.06) | 0.5 (0.06) |  | 0.5 (0.09) | 0.4 (0.08) | 0.5 (0.06) |  | 0.4 (0.07) | 0.4  (0.06) | 0.5 (0.06) |
| Most prevalent (%) |  |  |  |  |  |  |  |  |  |  |  |
| Hypertension | 11.7 | 10.4 | 12.7 |  | 15.4 | 13.9 | 16.6 |  | 11.1 | 9.8 | 12.1 |
| Chronic obstructive pulmonary disease | 6.6 | 5.9 | 7.2 |  | 7.6 | 6.8 | 8.2 |  | 7.2 | 6.4 | 7.8 |
| Diabetes | 4.9 | 4.3 | 5.3 |  | 6.7 | 5.9 | 7.3 |  | 6.3 | 5.6 | 6.8 |
| Depression | 4.1 | 4.0 | 4.2 |  | 2.0 | 1.9 | 2.2 |  | 2.5 | 2.3 | 2.6 |
| Fluid and electrolyte disorders | 2.2 | 2.1 | 2.3 |  | 1.7 | 1.6 | 1.7 |  | 1.7 | 1.7 | 1.7 |
| Cardiac arrythmia | 2.1 | 1.8 | 2.3 |  | 1.3 | 1.1 | 1.4 |  | 1.3 | 1.2 | 1.3 |

Notes: Table displays socioeconomic averages for insured ED visits for the full sample period (2011-2017) and stratified by pre- and post-ACA period.

Table S3: Difference-in-Differences and Difference-in-Differences-in-Differences Estimates of the Association of the ACA With the Uninsured ED Visit Rates by Race and Ethnicity (Excluding Massachusetts from the Treatment Group)

|  | **Pre-ACA** | **Post-ACA** | **Unadjusted** | **Combined** | **Adjusted ACA Only** | **Medicaid Expansion Only** |  |
| --- | --- | --- | --- | --- | --- | --- | --- |
|  |  |  |  |  |  |  |  |
| **Overall uninsured ED visits** | |  |  |  |  |  |  |
| White | 2222.7 | 2021.8 |  |  |  |  |  |
| Black | 4884 | 4342.1 | -341 | -589.6** | 33.2 | -622.8** |  |
| Hispanic | 2193.3 | 1950.5 | -41.9 | -25.5 | 183.6 | 209.1 |  |
| **Not Preventable/Injuries** | |  |  |  |  |  |  |
| White | 725.9 | 618.8 |  |  |  |  |  |
| Black | 1325.2 | 1134.1 | -84 | -202.6** | -21.2 | -181.4** |  |
| Hispanic | 680.4 | 578 | 4.7 | -30.5 | 61.3 | -91.8 |  |
| **PCT/Preventable** |  |  |  |  |  |  |  |
| White | 632.7 | 578.8 |  |  |  |  |  |
| Black | 1463.6 | 1271.2 | -138.5 | -164.4** | 27 | -191.4** |  |
| Hispanic | 635.6 | 560.2 | -21.5 | 5.2 | 59.3* | -54.1 |  |
| **Not Emergent** |  |  |  |  |  |  |  |
| White | 494.6 | 421.1 |  |  |  |  |  |
| Black | 1350.8 | 1152.6 | -124.7 | -167** | 6.7 | -173.7** |  |
| Hispanic | 547.5 | 459.8 | -14.2 | -26.6 | 44.9 | -71.5 |  |
| **Mental Health/Substance Use** | |  |  |  |  |  |  |
| White | 130.7 | 130.8 |  |  |  |  |  |
| Black | 173 | 175.2 | 2.1 | -10.6 | 12 | -22.5** |  |
| Hispanic | 95.6 | 89 | -6.7 | 4.6 | 5.1 | -0.5 |  |

Notes: Adjusted estimates were obtained from DD and triple differences coefficients using multivariable ordinary least squares regressions adjusted for sociodemographic and state-level covariates. The combined effect displays the impact of the national component of the ACA (column 5) and the Medicaid expansion (column 6). Each row represents estimates from a separate regression model in columns 4-6. Pre-ACA refers to the 2011 to 2013 years and post-ACA to the 2014 to 2017 years. DD: Difference-in-Differences, PCT: Primary Care Treatable. ** indicates statistically significant at the 1% level and * indicates statistically significant at the 5% level.

Table S4: Indirect Test of the Parallel Trends Assumption in the pre-ACA Implementation Period for all uninsured ED Visits Rates by Racial and Ethnic Groups

| **Overall uninsured ED visits** | Black-White gap | p-value | Hispanic-White gap | p-value |
| --- | --- | --- | --- | --- |
| Year-quarter |  |  |  |  |
| 2011/2 | -256.1 | 0.080 | -58.7 | 0.576 |
| 2011/3 | -295.8 | 0.074 | -59.9 | 0.463 |
| 2011/4 | -54.0 | 0.664 | 14.6 | 0.890 |
| 2012/1 | 66.9 | 0.537 | 4.1 | 0.966 |
| 2012/2 | -144.7 | 0.152 | -0.7 | 0.994 |
| 2012/3 | -175.9 | 0.171 | 46.0 | 0.646 |
| 2012/4 | -1.7 | 0.991 | 92.7 | 0.330 |
| 2013/1 | 77.4 | 0.418 | -65.4 | 0.605 |
| 2013/2 | -2.4 | 0.982 | -57.5 | 0.673 |
| 2013/3 | -4.7 | 0.973 | -30.1 | 0.841 |
| 2013/4 | 187.5 | 0.296 | -104.5 | 0.538 |

Notes: The above table displays the interaction term of year-quarter with the non-Hispanic black (or Hispanic) indicator in the pre-ACA period compared to non-Hispanic whites, with the first quarter in 2011 as the reference group. Data is limited to 2011-2013.

Table S5: Indirect Test of the Parallel Trends Assumption in the pre-ACA Implementation Period for Uninsured ED Visits Rates by Racial and Ethnic Groups Classifying Visits using NYU Algorithm

| **Not Preventable/Injuries** | Black-White gap | p-value | Hispanic-White gap | p-value | **PCT/Preventable** | Black-White gap | p-value | Hispanic-White gap | p-value |
| --- | --- | --- | --- | --- | --- | --- | --- | --- | --- |
| Year-quarter |  |  |  |  | Year-quarter |  |  |  |  |
| 2011/2 | -13.7 | 0.751 | -31.1 | 0.477 | 2011/2 | -142.0 | 0.021 | -9.0 | 0.812 |
| 2011/3 | -7.5 | 0.866 | 1.7 | 0.960 | 2011/3 | -188.0 | 0.001 | -25.4 | 0.421 |
| 2011/4 | 20.0 | 0.494 | 22.3 | 0.542 | 2011/4 | -64.2 | 0.212 | -10.7 | 0.767 |
| 2012/1 | 39.2 | 0.114 | 1.5 | 0.967 | 2012/1 | -25.1 | 0.610 | -12.2 | 0.725 |
| 2012/2 | 13.5 | 0.698 | -2.3 | 0.943 | 2012/2 | -118.6 | 0.010 | -10.5 | 0.771 |
| 2012/3 | 41.6 | 0.215 | 17.4 | 0.596 | 2012/3 | -167.7 | 0.003 | -16.2 | 0.652 |
| 2012/4 | 18.3 | 0.662 | 24.6 | 0.481 | 2012/4 | -32.5 | 0.629 | 12.5 | 0.753 |
| 2013/1 | 22.5 | 0.483 | -39.2 | 0.286 | 2013/1 | 5.0 | 0.926 | -21.4 | 0.683 |
| 2013/2 | 30.8 | 0.347 | -32.0 | 0.487 | 2013/2 | -77.6 | 0.137 | -32.3 | 0.494 |
| 2013/3 | 61.4 | 0.098 | -16.7 | 0.711 | 2013/3 | -124.8 | 0.052 | -38.2 | 0.469 |
| 2013/4 | 49.3 | 0.456 | -48.3 | 0.391 | 2013/4 | 16.2 | 0.776 | -48.2 | 0.427 |
|  |  |  |  |  |  |  |  |  |  |
| **Not Emergent** | Black-White gap | p-value | Hispanic-White gap | p-value | **Mental Health/Substance Use** | Black-White gap | p-value | Hispanic-White gap | p-value |
| Year-quarter |  |  |  |  | Year-quarter |  |  |  |  |
| 2011/2 | -77.9 | 0.064 | -18.1 | 0.520 | 2011/2 | -3.0 | 0.778 | 5.1 | 0.502 |
| 2011/3 | -87.2 | 0.077 | -12.0 | 0.547 | 2011/3 | -0.8 | 0.941 | 1.8 | 0.803 |
| 2011/4 | -16.9 | 0.622 | -2.9 | 0.916 | 2011/4 | 2.9 | 0.732 | 7.4 | 0.367 |
| 2012/1 | 29.9 | 0.321 | -3.8 | 0.893 | 2012/1 | 2.3 | 0.777 | 12.6 | 0.081 |
| 2012/2 | -38.0 | 0.167 | 2.8 | 0.917 | 2012/2 | -0.7 | 0.929 | 7.0 | 0.385 |
| 2012/3 | -54.0 | 0.091 | 17.1 | 0.537 | 2012/3 | -7.8 | 0.404 | 7.3 | 0.345 |
| 2012/4 | 9.4 | 0.823 | 25.6 | 0.353 | 2012/4 | -3.8 | 0.699 | 15.8 | 0.076 |
| 2013/1 | 43.6 | 0.123 | -11.9 | 0.746 | 2013/1 | -6.9 | 0.471 | 16.4 | 0.076 |
| 2013/2 | 25.3 | 0.409 | -2.0 | 0.958 | 2013/2 | -5.1 | 0.568 | 6.9 | 0.460 |
| 2013/3 | 18.1 | 0.609 | 2.7 | 0.950 | 2013/3 | -1.4 | 0.876 | 7.1 | 0.462 |
| 2013/4 | 83.0 | 0.081 | -19.0 | 0.680 | 2013/4 | -3.6 | 0.772 | 8.2 | 0.443 |

Notes: See Table S4.
